# Supplementary material for: Model-based evaluation of school- and non-school-related measures to control the COVID-19 pandemic
Source: Nat Commun. 2021 Mar 12;12:1614. doi: 10.1038/s41467-021-21899-6 (PMC7955041; doi:10.1038/s41467-021-21899-6)
Supplement: Supplementary file 1 — Supplementary Information [file 41467_2021_21899_MOESM1_ESM.pdf]

Supplementary Information

Model-based evaluation of school- and non-school-related measures to control the COVID-19 pandemic

Ganna Rozhnova, PhD*[1,2], Christiaan H. van Dorp, PhD[3], Patricia Bruijning-Verhagen, MD PhD[1], Martin C.J. Bootsma, PhD[1,4], Prof Janneke H.H.M. van de Wijgert, MD PhD MPH[1,5], Prof Marc J.M. Bonten, MD PhD[1,6], and Prof Mirjam E. Kretzschmar, PhD[1]

[1]Julius Center for Health Sciences and Primary Care, University Medical Center Utrecht, Utrecht University, Utrecht, The Netherlands

[2]BioISI—Biosystems & Integrative Sciences Institute, Faculdade de Ciências, Universidade de Lisboa, Lisboa, Portugal

[3]Theoretical Biology and Biophysics (T-6), Los Alamos National Laboratory, Los Alamos, New Mexico, USA

[4]Mathematical Institute, Utrecht University, Utrecht, The Netherlands

[5]The Institute of Infection, Veterinary and Ecological Sciences, University of Liverpool, Liverpool, UK

[6]Department of Medical Microbiology, University Medical Center Utrecht, Utrecht University, The Netherlands

February 14, 2021

*Corresponding author:

Dr. Ganna Rozhnova

Julius Center for Health Sciences and Primary Care

University Medical Center Utrecht

P.O. Box 85500 Utrecht

The Netherlands

Email: g.rozhnova@umcutrecht.nl

Phone: +31 683890206

Computation of the basic and effective reproduction numbers

There are several methods that can be used to calculate the basic reproduction number (in the absence of interventions), R_0, and the effective reproduction number (in the presence of interventions), R_e, for a pathogen (see e.g. [1]). We used the next-generation matrix method which is discussed for compartmental epidemiological models in [2–4], and which we applied previously to HIV [5] and CMV models [6]. We first briefly revise the steps of the calculation of R_0 for our model given by Eq. 4 and Eq. 5 (both from the main text), and then describe our procedure for calculating R_e.

Calculation of R_0

The steps in the calculation of R_0 are

1. The starting point is the system of ordinary differential equations, Eq. 4 (main text), without control measures. This means that the contact rate $c_{kl}(t)$ in Eq. 5 (main text) is substituted by the contact rate before the first lockdown, b_{kl}, as follows

$$\lambda_k(t) = \epsilon \sum_{l=1}^{n} \sum_{p=1}^{m} b_{kl} \frac{I_{l,p}(t)}{N_l}.$$

2. Calculate the Jacobian matrix, $\mathbf{J}$, of Eq. 4 (main text).

3. Evaluate the Jacobian, $\mathbf{J}$, at the infection free equilibrium

$$S_l^* = N_l, \quad E_l^* = I_{l,p}^* = H_l^* = R_l^* = 0, \quad p = 1, \ldots, m, \quad l = 1, \ldots, n.$$

4. As demonstrated in e.g. [3], the Jacobian, $\mathbf{J}$, can be written as a sum of two matrices, the matrix of transmissions $\mathbf{T}$ and the matrix of transitions $\mathbf{\Sigma}$

$$\mathbf{J} = \mathbf{T} + \mathbf{\Sigma}.$$

   The matrix of transmissions, $\mathbf{T}$, contains elements of $\mathbf{J}$ proportional to the probability of transmission per contact, ϵ. The matrix of transitions, $\mathbf{\Sigma}$, contains the remaining elements of $\mathbf{J}$, $\mathbf{\Sigma} = \mathbf{J} - \mathbf{T}$.

5. R_0 then equals the dominant eigenvalue of the next generation matrix $\mathbf{K}$ defined as follows [3]

$$\mathbf{K} = -\mathbf{T}\mathbf{\Sigma}^{-1}.$$

The explicit expressions for matrices $\mathbf{J}$, $\mathbf{T}$, $\mathbf{\Sigma}$ and $\mathbf{K}$ are given in the Mathematica notebook SchoolAnalyses.nb available in the GitHub repository, https://github.com/lynxgav/COVID19-schools. Supplementary Figure 4 **a** was obtained following the described procedure using 2000 parameter samples from the posterior distribution.

Calculation of R_e from February 23 till April 30

The calculation of R_e follows the same steps, with the only difference that the starting point is the system of ordinary differential equations, Eq. 4 (main text), with control measures. The parameters describing epidemiology of SARS-CoV-2 (e.g. probability of transmission per contact, infectious period, latent period, hospitalization rate) are assumed not to be affected by control measures. Therefore, most of the estimated parameters (Table 2) are constant throughout the time horizon of the analyses which spans both pre-lockdown, post-lockdown and relaxation periods. Keeping basic epidemiological parameters fixed is a natural choice since they describe the biology of the virus which should not be affected by control measures. The hospitalization rate could, in principle, be varying with time, but this is not the case in the Netherlands where the criteria for hospital admission have not changed during the pandemic (see Section Data in the main text for more details). The time-dependent parameter is the contact rate $c_{kl}(t)$ given by Eq. 1 (main text) for the period from before to after the first lockdown and by Eq. 3 (main text) for the relaxation period after the first lockdown. Additionally, we allow for different reduction in probability of transmission per contact in the post-lockdown (ζ_1) and relaxation (ζ_2) periods due to general population-based measures that are not explicitly included in the model (e.g. mask-wearing and self-isolation of symptomatic persons). For the post-lockdown period, the contact rate $c_{kl}(t)$ is substituted by the contact rate after the first lockdown, a_{kl}, and ϵ is substituted by $\epsilon\zeta_1$. Then Eq. 5 (main text) becomes

$$\lambda_k(t) = \epsilon\zeta_1 \sum_{l=1}^{n}\sum_{p=1}^{m} a_{kl}\frac{I_{l,p}(t)}{N_l}.$$

Supplementary Figure 4 **b** was obtained following the described procedure using 2000 parameter samples from the posterior distribution. Calculation of R_e at the intermediate points is done similarly, where Eq. 5 (main text) is given by

$$\lambda_k(t) = \epsilon \sum_{l=1}^{n}\sum_{p=1}^{m}\left[\left(1-\frac{1}{1+e^{-K_1(t-t_1)}}\right)b_{kl} + \frac{1}{1+e^{-K_1(t-t_1)}}\zeta_1 a_{kl}\right]\frac{I_{l,p}(t)}{N_l}.$$

Note that while calculating R_e at times $t > 0$, the Jacobian, $\mathbf{J}$, of Eq. 4 (main text), was evaluated at the infection free equilibrium in the same way as we did for R_0. This is a frequently used procedure that was applied for a number of pathogens, including SARS-CoV-2 (see e.g. recent study by Li et al [7]). If the decrease in the prevalence of susceptible persons was taken into account, the estimated R_e would be lower, but the corrections to R_e would be small and would not change the overall conclusions of our study which is focused on the relative impact of school- and non-school-based measures. The reason for this is that the seroprevalence of SARS-CoV-2 in the general population in the Netherlands is very low during the time horizon of our analyses. It was measured at 2.8%, 4.6% and 4.9% in 3 rounds of the national serological survey conducted in April/May, June/July and September/October 2020 (https://www.rivm.nl/pienter-corona-studie/resultaten). Using the age-specific seroprevalence estimates from the 3 serosurvey rounds we obtain R_e for Round 1 0.60 (95%CrI 0.28—0.72) (2.9% correction for the median

R_e in Supplementary Figure 4 **b**), for Round 2 1.25 (95%CrI 1.10—1.98) (4.7% correction for the mean R_e in Supplementary Figure 4 **c**), and for Round 3 0.96 (95%CrI 0.89—1.27) (4.6% correction for the median R_e in Supplementary Figure 4 **d**). In all cases, the corrections are much smaller than the credible intervals of our estimates.

Calculation of R_e after April 30

Reliable hospitalization data during relaxation is not available because hospitals in the Netherlands are not anymore required to report admissions with COVID-19 to OSIRIS database we used in this study. For August and November 2020 (Supplementary Figures 4 **c** and **d**), we calibrated the model to values of R_e as published on the dashboard of the National Institute for Public Health and the Environment (RIVM) [8]. Methods for the estimation of these time-dependent R_e values are described in [9]. The calibration of the model in the relaxation period is possible because, as described above, the epidemiological parameters are assumed to be constant throughout the time horizon of the analyses, and only the contact structure as described by g, ω and ζ_2 (constant during the relaxation period) varies with time (see Eq. 3, main text). Since, in the relaxation period, schools were open without substantial control measures, ω was fixed at 1 (the proportion of retained school contacts during the relaxation period as compared to the pre-lockdown period). Since there was some decrease in adherence to contact-reduction measures in August and November as compared to April, ζ_2 was fixed at 0.67 (ζ_1 is estimated at 0.51 for April, Supplementary Figure 3). We finally calibrated g such that the median reproduction numbers in the model would equal the specific values estimated by the RIVM (about 1.3 in the period 27 August-6 September and about 1 in the period 7-13 November) [8]. Parameter g can be easily interpreted using Eq. 3 (main text). For example, $g = 0.5$ corresponds to half-way in the relaxation of non-school contacts. The distributions shown in Supplementary Figures 4 **c** and **d** are obtained using the NGM method with $\omega = 1$, $\zeta_2 = 0.67$, $g = 0.5$ and $g = 0.8$, respectively, and other parameters drawn from the posterior distributions as shown in Supplementary Figure 3.

References

[1] Vynnycky E, White R. An introduction to infectious disease modelling. Oxford: Oxford University Press; 2010.

[2] van den Driessche P, Watmough J. Reproduction numbers and sub-threshold endemic equilibria for compartmental models of disease transmission. Mathematical Biosciences. 2002;180:29–48.

[3] Diekmann O, Heesterbeek JAP, Roberts MG. The construction of next-generation matrices for compartmental epidemic models. Journal of The Royal Society Interface. 2010;7(47):873–885. doi:10.1098/rsif.2009.0386.

[4] Diekmann O, Heesterbeek H, Britton T. Mathematical Tools for Understanding Infectious Disease Dynamics. Princeton University Press; 2013.

[5] Rozhnova G, van der Loeff MFS, Heijne JCM, Kretzschmar ME. Impact of heterogeneity in sexual behavior on effectiveness in reducing HIV transmission with test-and-treat strategy. PLOS Computational Biology. 2016;12(8):e1005012. doi:10.1371/journal.pcbi.1005012.

[6] Rozhnova G, Kretzschmar ME, van der Klis F, van Baarle D, Korndewal M, Vossen AC, et al. Short- and long-term impact of vaccination against cytomegalovirus: a modeling study. BMC Med. 2020;18. doi:https://doi.org/10.1186/s12916-020-01629-3.

[7] Li R, Pei S, Chen B, Song Y, Zhang T, Yang W, et al. Substantial undocumented infection facilitates the rapid dissemination of novel coronavirus (SARS-CoV-2). Science. 2020;368(6490):489–493. doi:10.1126/science.abb3221.

[8] Coronavirus dashboard; 2020. Available from: https://coronadashboard.government.nl/.

[9] Wallinga J, Lipsitch M. How generation intervals shape the relationship between growth rates and reproductive numbers. Proceedings of the Royal Society B: Biological Sciences. 2007;274(1609):599–604. doi:10.1098/rspb.2006.3754.

Supplementary Figures

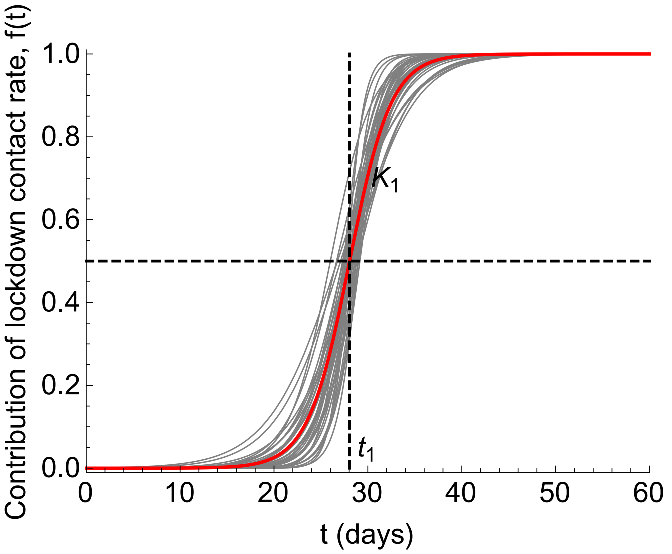

Supplementary Figure 1. Contribution of the contact rate after the first lockdown. We model the transition in the general contact rate, $c_{kl}(t)$, as follows $c_{kl}(t) = [1 - f(t)]b_{kl} + \zeta_1 f(t) a_{kl}$, where $f(t)$ is the contribution of the contact rate after the first lockdown, b_{kl} and a_{kl} are the contact rates specific to the periods before and after the first lockdown. $f(t)$ is a logistic function with parameters K_1 and t_1 governing the speed and mid-way of lockdown roll-out. The red and gray lines show the median and several individual estimated trajectories, respectively.

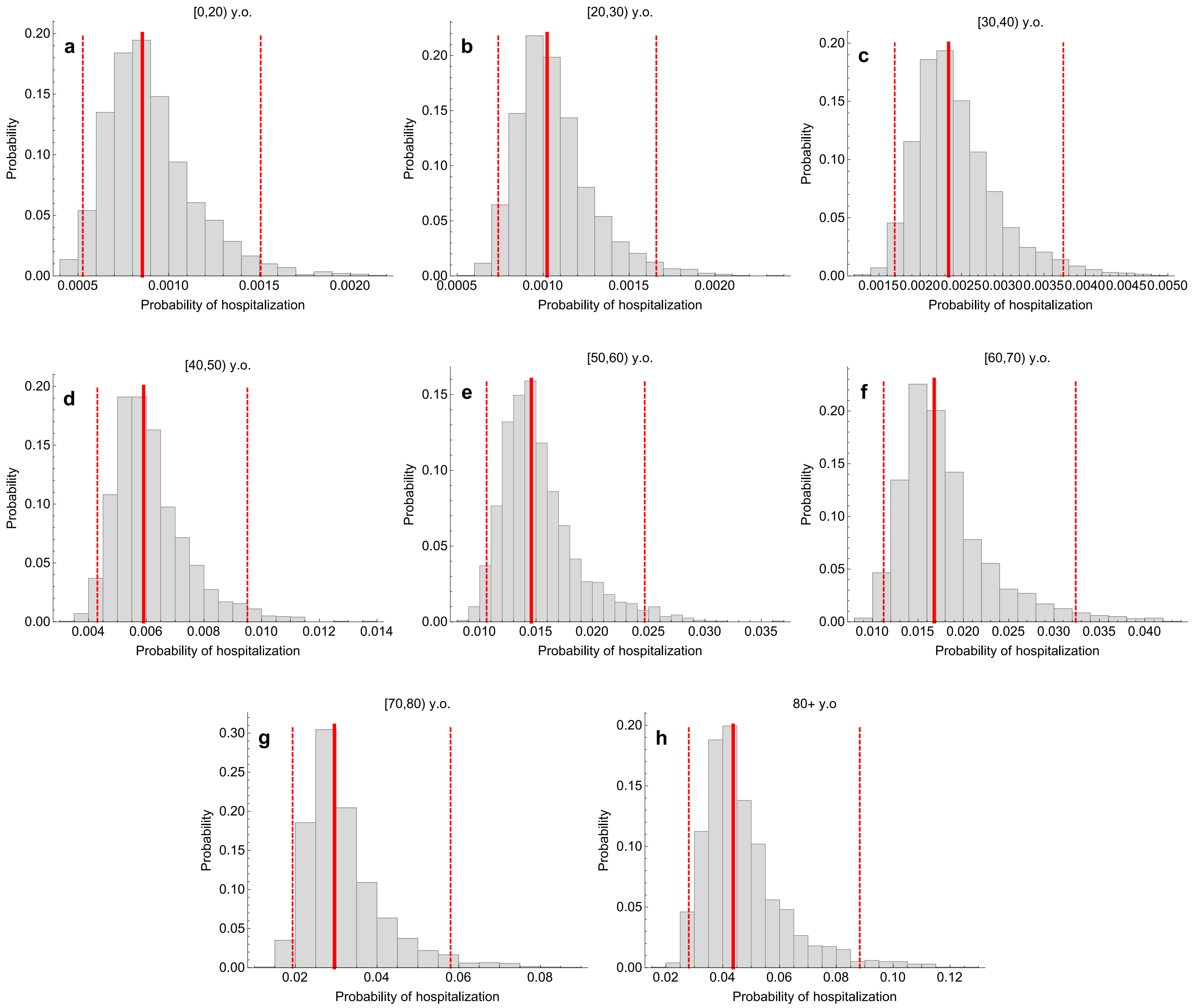

Supplementary Figure 2. Estimates of probabilities of hospitalization. Histograms are based on 2000 parameter samples from the posterior distribution. The solid and the dashed lines correspond to the median and 95% credible intervals. Panels **a-h** refer to different age groups.

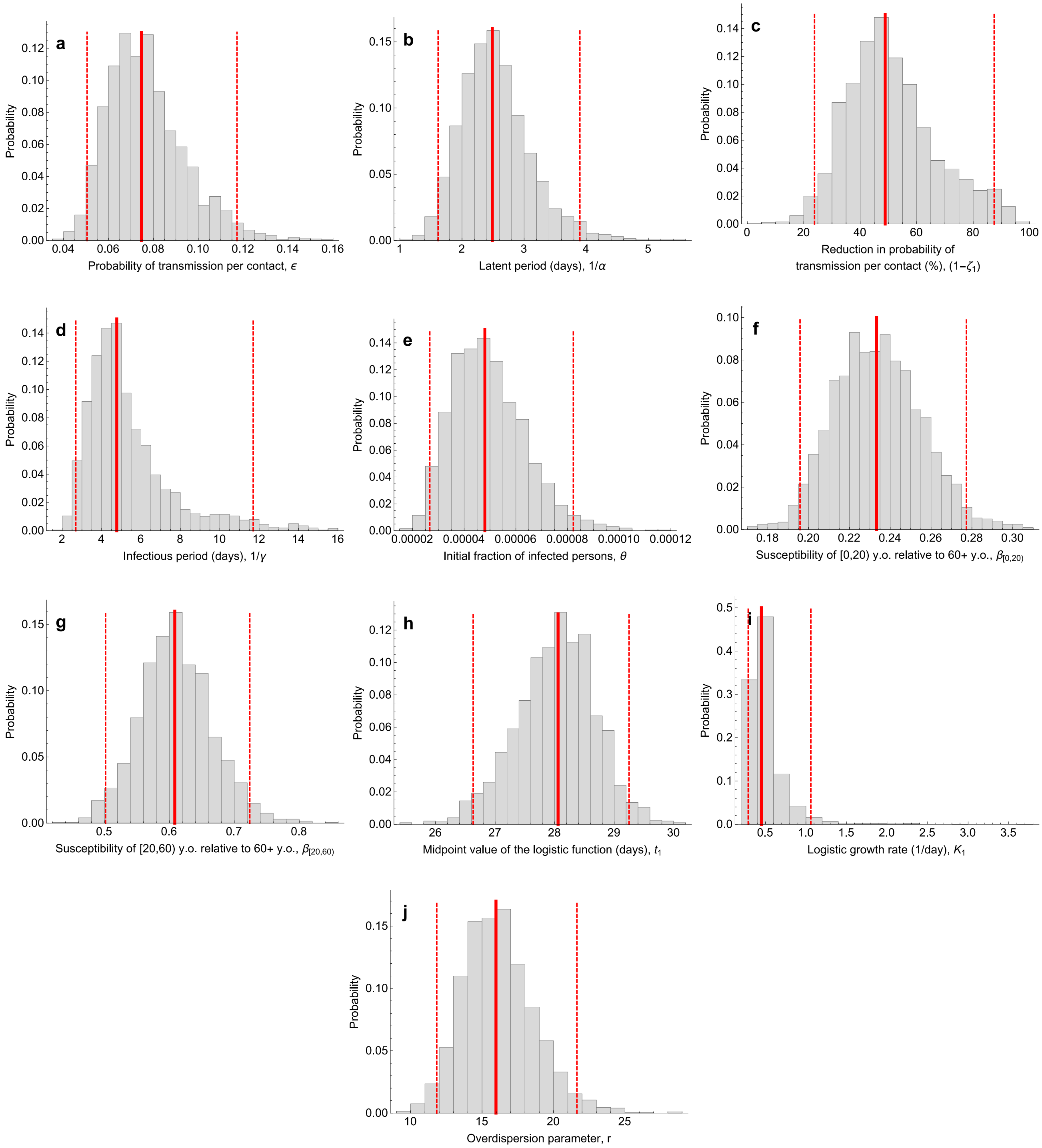

Supplementary Figure 3. Parameter estimates. Histograms are based on 2000 parameter samples from the posterior distribution. The solid and the dashed lines correspond to the median and 95% credible intervals. Panels **a-j** refer to different parameters from Table 1 in the main text.

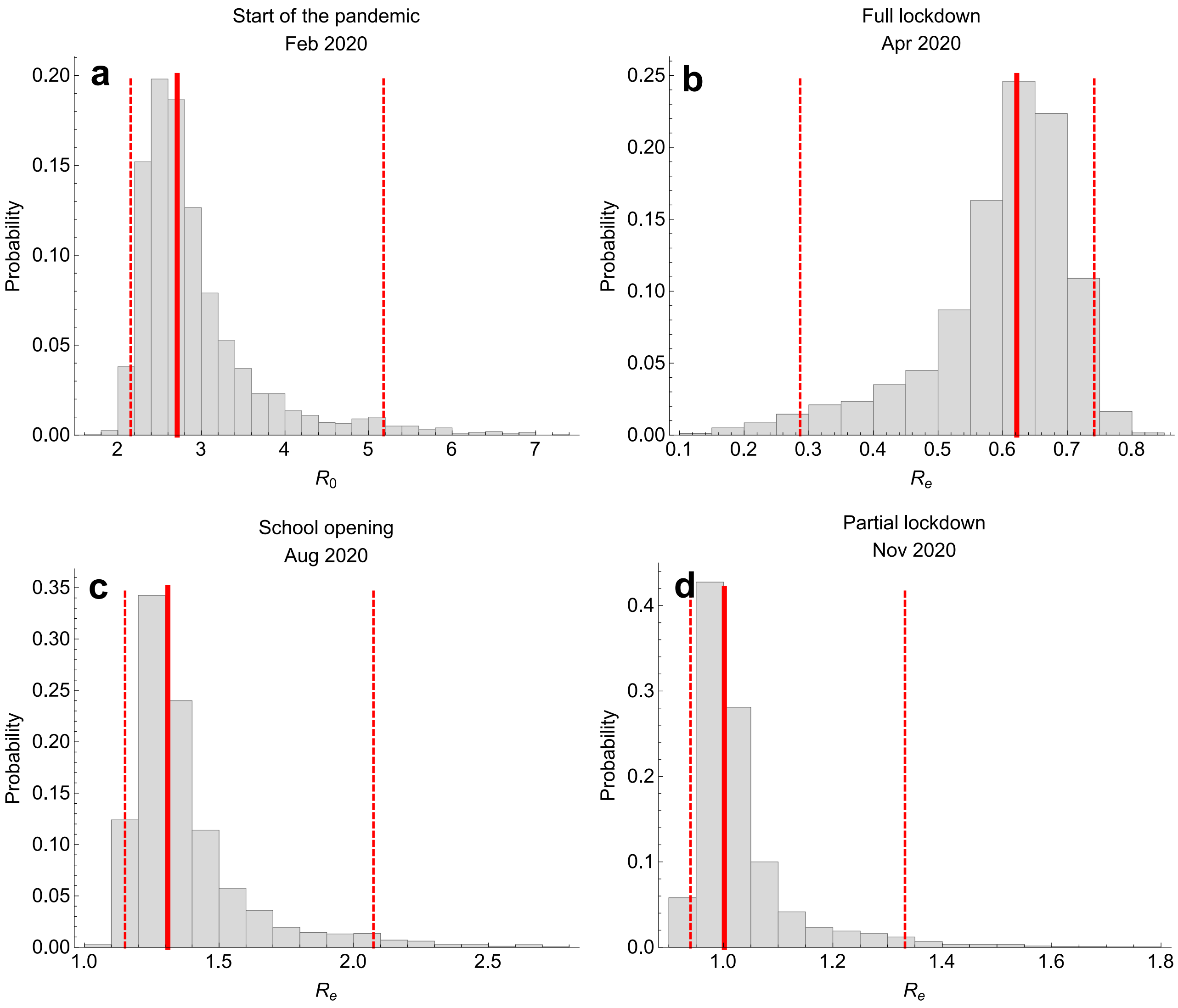

Supplementary Figure 4. Reproduction numbers. Estimated reproduction numbers **a** at the beginning of the pandemic (February 2020), **b** after the first full lockdown (April 2020), **c** at the time of school opening (August 2020), and **d** after the second partial lockdown (November 2020). Histograms are based on 2000 parameter samples from the posterior distribution. The solid and the dashed lines correspond to the median and 95% credible intervals.

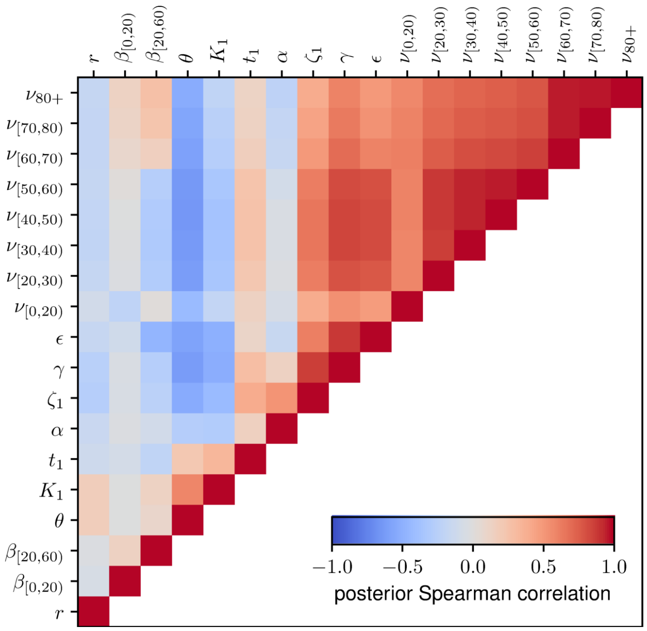

Supplementary Figure 5. Correlation matrix. Correlation matrix for 10 parameters estimated in the model. As hospitalization rate and susceptibility are age-dependent parameters, 18 numbers have been estimated in total. The color denotes posterior Spearman correlation.
